# Quantitative and qualitative analysis of quality of life in people diagnosed with genetic haemochromatosis in the United Kingdom

**DOI:** 10.1186/s41687-025-00865-6

**Published:** 2025-07-29

**Authors:** Leah Craven-Smith, Neil McClements, Diogo Gomes, Victoria Pointon

**Affiliations:** 1 University of Leeds, Leeds, England; 2 Haemochromatosis UK, Spalding, England; 3 Advanced Nurse Practitioner - Information & Advisory, Haemochromatosis UK, Spalding, England; 4 Information & Advisory, Haemochromatosis UK, Spalding, England

**Keywords:** Genetic haemochromatosis, Quality of life, WHOQOL-100, Physical symptoms, Psychological symptoms, QoL, Hereditary haemochromatosis, Genetics

## Abstract

**Background:**

Genetic haemochromatosis (GH) is a long-term genetic condition which results in increased iron absorption into the blood and accumulation of iron into certain organs overtime. Increased absorption and accumulation can be fatal. GH can cause many symptoms including arthritis/joint pain, chronic fatigue, and cognitive difficulties. The aim of this study was to measure quality of life (QoL) in people diagnosed with GH (GH-diagnosed) compared to a healthy sample and identify possible explanations for this.

**Methodology:**

QoL was measured in 535 healthy people and 1039 GH-diagnosed, through completion of the World Health Organisation Quality of Life-100 survey (WHOQOL-100). 985 GH-diagnosed respondents completed a GH-focussed survey, which was developed to get further details of the impact of GH.

**Results:**

Comparison of the WHOQOL-100 overall QoL score between GH-diagnosed and the healthy sample found a significantly lower score in the GH-diagnosed. Physical, psychological, level of independence, and spiritual domains were significantly lower in the GH-diagnosed group. The GH-focussed survey found a high incidence of physical and mental symptoms, and some impact on social and work life. Areas in which participants suggest would improve their QoL included: improved healthcare especially with increased understanding of GH in medical professionals, increased access to appointments, in-person appointments, regular checks for organ damage, more nutrition or dietary advice, and local support groups.

**Conclusions:**

Based on the WHOQOL-100 scores and GH-focussed survey, overall QoL is worse in people diagnosed with GH due to worse physical and psychological symptoms. Improved healthcare may aid in reducing the difference in QoL.

## Background

Genetic haemochromatosis (GH) is a common long-term autosomal recessive disorder [1], which causes increased iron absorption, transferrin saturation, and iron accumulation in organs overtime [2–4]. Type 1, 2, 3, and 4 are the different forms of GH, each characterised by different genetic mutations and often have differing phenotypic expressions [5–8]. Type 1 haemochromatosis is the most common and is characterised by homozygous C282Y, homozygous H63D, or heterozygous C282Y/H63D mutations in the HFE gene [1]. The homozygous C282Y mutation is the most commonly diagnosed genotype, usually with the worst phenotypic expression, and seen in approximately 1 in 150 Northern Europeans [1, 5, 8–10].

GH has been shown to express a range of symptoms and associated conditions including: pituitary dysfunction, diabetes, hairloss, menstrual problems, breathing or heart difficulties, abdominal pain, skin problems, sexual health issues, psychological or cognitive difficulties, fatigue [11], arthritis or joint pain [12–15], dermatological issues [16, 17], fibromyalgia [18], gut problems [19]. hypothyroidism [16], and increased susceptibility to infection [20]. Symptoms and diagnosis of HFE (type 1) GH usually occur after the age of 40 [1, 2, 21], but overload and adverse effects have been shown to begin at childhood and adolescence [22]. Iron accumulation occurs in the heart, liver, pancreas, and gonads [23, 24]. Iron accumulation in the liver can result in fibrosis and cirrhosis, leading to hepatocellular carcinoma and accumulation in the heart can result in cardiomyopathy [24]. GH causes increased cancer risk [25] and iron accumulation can result in mortality [24].

GH is usually treated by venesection to reduce blood iron levels and maintain an iron level of approximately 50 µg/l [26]. There is increasing interest in erythrocytapheresis, chelators, hepcidin peptide mimetics, and proton-pump inhibitors as alternative treatments [19, 26–28]. Early diagnosis and intervention is important to reduce irreversible organ damage and improve patient outcomes [29]. Despite treatment to allow iron levels to reach normal therapeutic levels and reduce organ accumulation, GH symptoms can persist and there is a lack of evidence to show reversal of diabetes and hepatocellular carcinoma following venesection [30].

Quality of life (QoL) is defined by the world health organisation (WHO) as an ‘*individuals’ perception of their position in life in the context of the culture and value systems in which they live and in relation to their goas*,* expectations*,* standards and concerns’* [31]. The World Health Organisation Quality of Life-100 (WHOQOL-100) survey assesses QoL using scoring of 24 facets and 6 domains. These domains are physical, psychological, level of independence, social relationships, environment, and spirituality. WHOQOL-100 has been found to be valid and reliable for measurement of QoL in the united states, china, and the Netherlands, in healthy people, childbearing women, chronically ill adults, carers, psychiatric outpatients, patients with schizophrenia, and patients diagnosed with breast cancer [32–36]. Relevant to this study, WHOQOL-100 has been validated for use in Britain for sick and healthy respondents, and to assess antidepressant outcomes in primary care [37, 38]. These studies found good overall discriminatory power, overall consistency reliability (Cronbach’s α = 0.967 and 0.96) [37, 38]. Overall, high construct, convergent, and divergent validity was seen in WHOQOL-100 domains and facets [38].

There is a lack of studies comparing the QoL of people diagnosed with GH (GH-diagnosed) compared to a healthy sample, specifically in the UK and utilising WHOQOL-100. However, QoL of chronic liver patients diagnosed with GH was assessed using the Liver Disease Symptom Index, Short Form-36 (SF-36), and Multi-Dimensional Fatigue Index-20 surveys, to find a significantly worse QoL when compared to a healthy Dutch control population [39]. Additionally, QoL of GH-diagnosed in Australia was measured using the Assessment of Quality-of-Life 4D and found a decrease in scores compared to the previously published normative data [29]. QoL of GH-diagnosed was previously measured using the SF-36 survey to compare QoL between the different genotypes of GH; a worse QoL was identified in those with a homozygous C282Y mutation compared to other genotypes [8]. Fonseca et al., (2018) also confirmed these findings [5]. A study across a range of countries, found research into arthritis and joint problems, promotion of knowledge about GH among medical doctors, and investigation of new or alternative treatments for GH, were the top priorities believed to improve QoL in GH-diagnosed [19]. This suggests that these areas contribute in the reduction of QoL in GH-diagnosed.

The aim of this study is to compare QoL between GH-diagnosed and a healthy sample using quantitative data from the WHOQOL-100 survey, and to identify possible reasons or themes using a survey specific to GH-diagnosed. The hypothesis of this study is that QoL will be significantly less in GH-diagnosed compared to a healthy sample.

## Methodology

### Participants

Data was collected from 12th June to 6th August 2024. Data for WHOQOL-100 and GH-focussed survey was collected from GH-diagnosed, by random sampling and online advertisement, with the survey advertised to Haemochromatosis UK (HUK) members and the larger GH community through social media. Responses for the WHOQOL-100 survey, from GH-diagnosed, were collected from 1131 participants and responses from under 18’s or falsified birthdays were removed to leave 1039 respondents. Sex and age distribution was determined to allow matching of a healthy control group. Responses were collected from 1416 participants, through advertisement for completion through the website SurveySwap. Responses from people diagnosed with long-term health conditions, those who failed attention checks and duplicates were removed to leave 535 healthy WHOQOL-100 respondents. Responses to the GH-focussed survey were collected from 1160 participants, with responses removed from people not diagnosed with GH to leave 985 respondents.

Consent: Respondents were informed that their responses would be used to assess QoL in people with GH and their data would be used in line with the HUK data privacy and protection policy.

### The WHOQOL-100 survey

The WHOQOL-100 survey consists of 100 questions completed by the participant. It contains six domains, and twenty-four facets to give a measure of the quality of different aspects of life and can give an overall QoL score. Each question requires the participant to provide a score using the 5-point Likert scale. Mean scores were calculated for each domain and facet. Higher scores indicate better QoL. Participants had unlimited time to complete the WHOQOL-100 questionnaire, but GH-diagnosed took an average of 25 min to complete and the healthy sample 16 min. The use of WHOQOL-100 was approved by the WHO Permissions team.

### GH-focussed survey

The GH-focussed survey was designed by initial scoping of the literature and use of frequently reported experiences of living with GH, with questions specifically targeted to GH-diagnosed. The survey was an online self-assessment designed to investigate QoL through questions about physical health, emotional wellbeing, treatment, social and work life, and disease specific concerns. Questions in the survey contained a mix of Likert scale, multiple choice response or open-text responses. Participants had unlimited time to complete the questionnaire but took an average of 17 min.

### Statistical analysis

Responses were reported as percentages of response or as mean values. Shapiro-Wilks test was used to determined data distribution. Comparative analysis of healthy and GH-diagnosed WHOQOL-100 respondents were performed using Mann-Whitney U test and Kruskal-Wallis Test with Dunn’s post hoc tests where appropriate. The level of significance was set as *p* < 0.05. Analysis was performed using Microsoft Excel Version 16.87, and IBM SPSS Statistics version 29.0.

## Results

### Characteristics of participants

Sociodemographic characteristics and GH genotypic distribution for WHOQOL-100 and GH-focussed survey respondents are shown in Table 1. GH-diagnosed and healthy WHOQOL-100 respondents, had mean ages of 59.00 ± 0.36 and 57.32 ± 0.57 respectively. Age group distribution was significantly different (*p* < 0.05) upon comparison of participants of the healthy sample WHOQOL-100 and GH-focussed surveys with GH-diagnosed WHOQOL-100 respondents. Sex distribution was not significantly different (*p* = 0.908). 47.0% of GH-diagnosed and 45.6% healthy WHOQOL-100 respondents, were university or college graduates. Significant differences in marital status, education, country of residence, and income were seen. Significantly, lower levels of education, income and percentage of married respondents were seen in the healthy sample.

For both GH surveys, the majority of participants were diagnosed with C282Y/C282Y homozygous GH and diagnosed between 51 and 60 years old. Most respondents were not in a maintenance phase, with serum ferritin over 100 mcg/l and transferrin saturation over 50%. Genotypic distribution (*p* = 0.485), age of diagnosis (*p* = 0.095), and maintenance (*p* = 0.546) were not significantly different between the two GH-diagnosed samples.

**Table 1:**
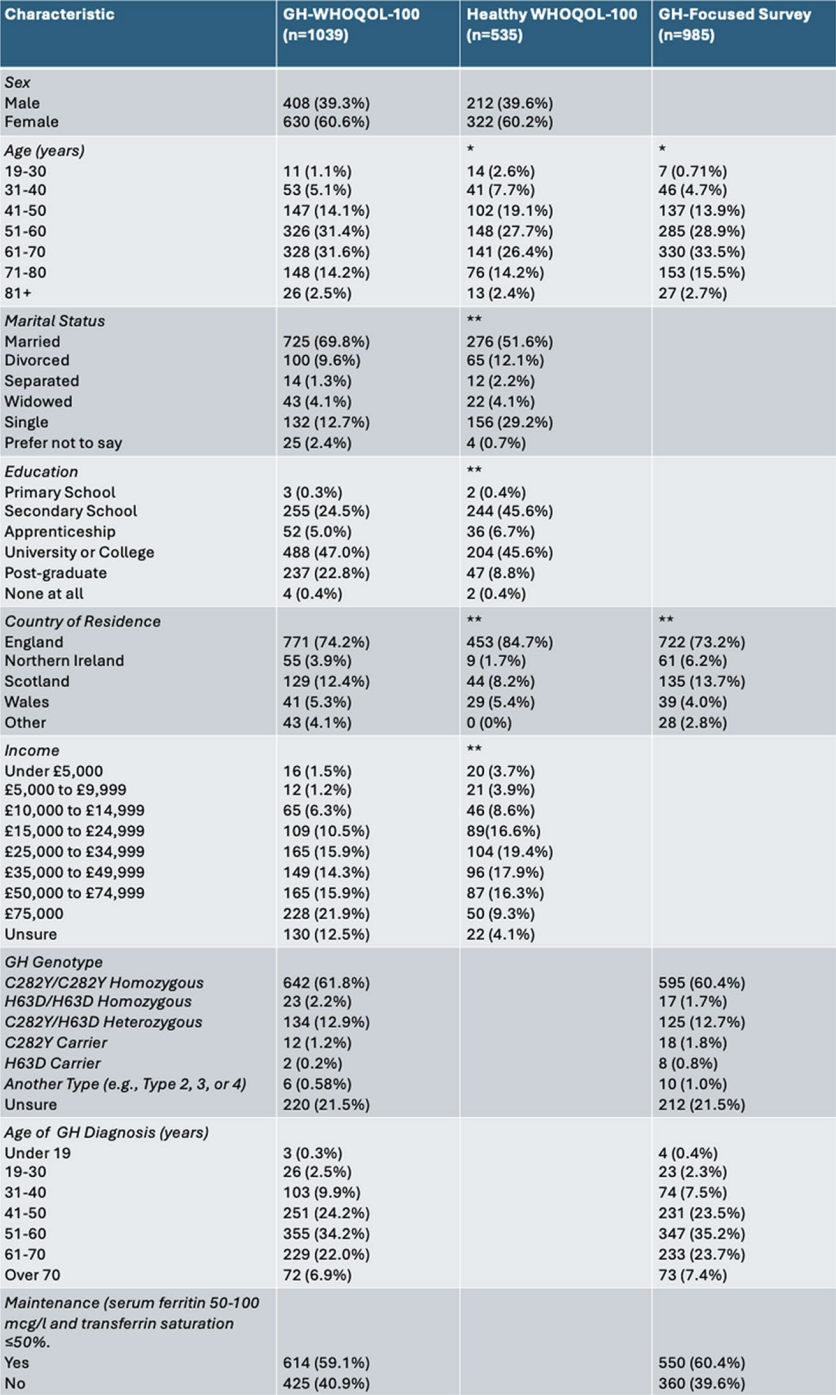
Sociodemographic characteristics of the genetic haemochromatosis (GH) and healthy sample who completed WHOQOL-100, and GH-diagnosed Poplution who completed the GH-focussed survey. Classification of GH genotype, age of diagnosis, and maintenance for respondents diagnosed with GH. *p < 0.05 and **p < 0.001 when GH-diagnosed WHOQOL-100 respondents, healthy WHOQOL-100 respondents, GH-focussed surved compared using Mann-Whitney U test and Kruskal-Wallis test with Dunn’s post hoc tests

From the 1039 GH-diagnosed WHOQOL-100 respondents, 45.9% of these were diagnosed with another long-term illness/condition. The most common conditions are listed in Table 2. Other conditions reported which were slightly less common included: migraine/headache (1.0%), spinal issues (1.3%), celiac (0.6%), and skin (1.2%), eye (0.9%), kidney (1.6%), and bladder (0.6%) conditions.

**Table 2:**
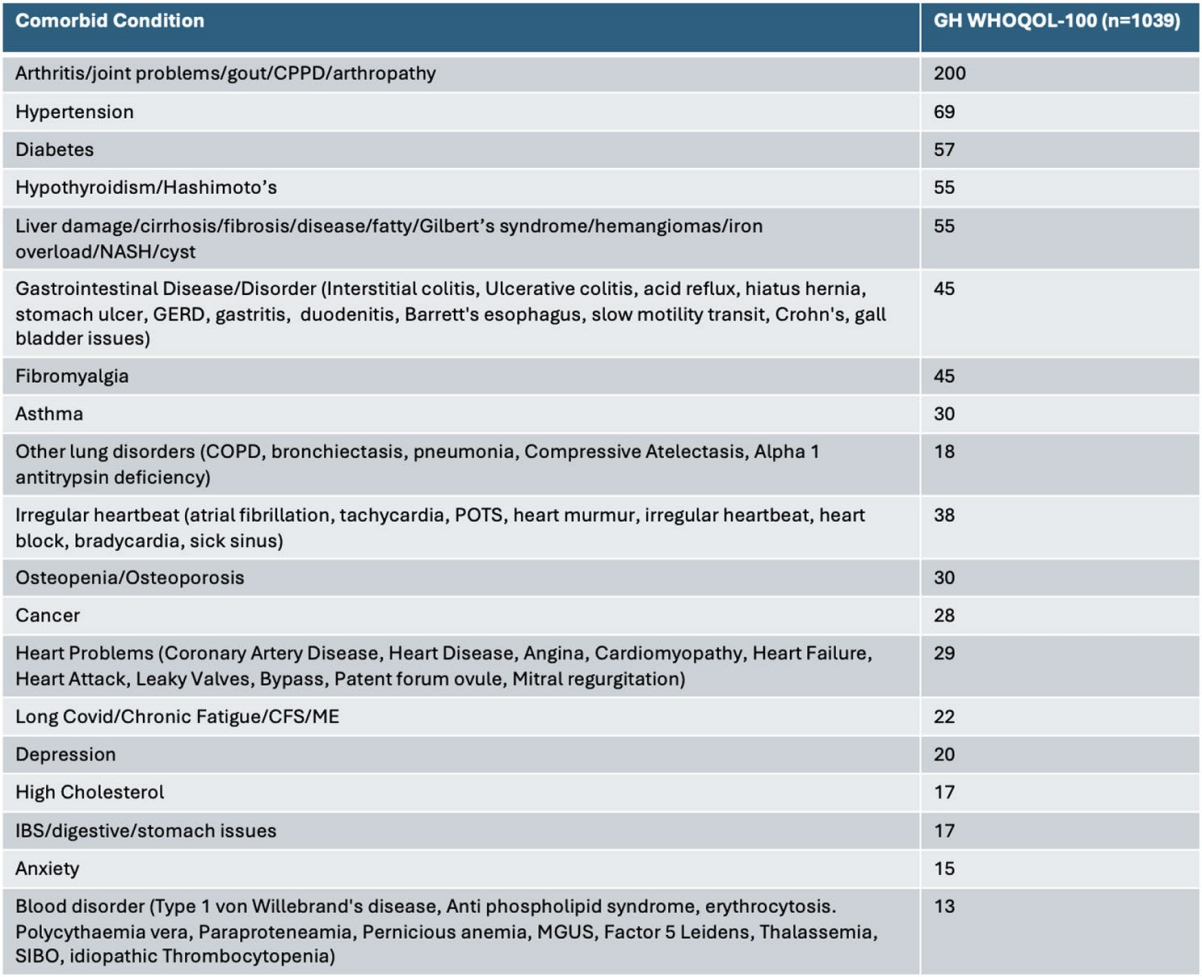
Number of respondents who are diagnosed with each of the conditions listed

### WHOQOL-100

Table 3 shows the overall QoL, domain, and facet scores. The overall QoL score in GH-diagnosed of 13.6 ± 0.11 was significantly lower than the score of 14.6 ± 0.15 in the healthy respondents (*p* < 0.001). GH-diagnosed also had significantly lower scores in physical, psychological, level of independence, and spiritual domains (*p* < 0.05). However, the environment domain was significantly greater in the GH-diagnosed respondents (*p* = 0.025). There was no significant difference between the social relationships’ domain (*p* = 0.124). Additionally, the QoL score in GH-diagnosed was significantly higher in those in maintenance compared to those not in maintenance, with scores of 13.8 ± 0.14 and 13.2 ± 0.18 respectively. Both of these scores were significantly less than the healthy control (*p* < 0.001).

Facets 2, 3, 5, 6, 9, 10, 12, 15, 19, 21, and 24 were significantly lower in the GH-diagnosed sample compared to the healthy control (*p* < 0.05). However, facets 1, 8, 11, 14, 16, 17, 18, 22, and 23 were significantly higher in GH-diagnosed (*p* < 0.05). The majority of the higher scores in the GH-diagnosed, were in the environment domain, excluding health/social care (F19), opportunities for recreation (F21), and spirituality (F24). Facets 4, 7, 13, and 20 were not significantly different between the two samples (*p* > 0.05).

**Table 3:**
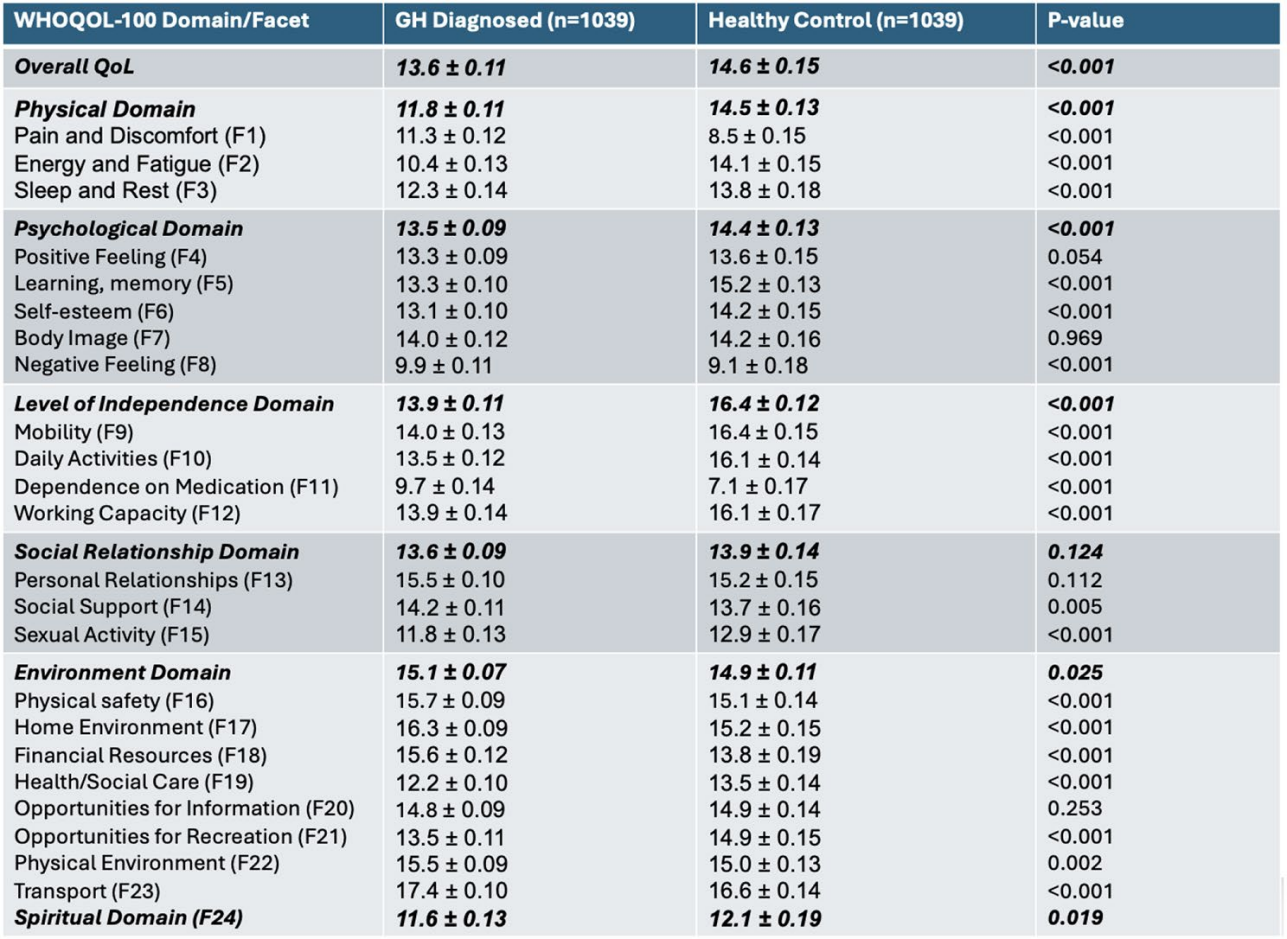
WHOQOL-100 scores for overall quality of life, the six domains, and all 24 facets (means ± SEM) for people diagnosed with genetic haemochromatosis and the healthy sample

### GH-focussed survey

#### Physical health

Overall, physical health was rated as 3.3 ± 0.03 out of 5; a score of 3 means neither poor nor good. This rating of physical health was significantly greater in those in maintenance compared to those not in maintenance, with mean scores of 3.34 ± 0.04 and 3.17 ± 0.05 respectively (*p* = 0.0072).

96.4% of respondents reported at least one physical symptom since diagnosis of GH. Table 4 shows the percentage of respondents who have experienced each of the physical symptoms listed; the top three symptoms experienced were arthritis/joint pain, chronic fatigue, and brain fog. These same symptoms had the biggest impact on daily activities. Other physical symptoms reported were dizziness/vertigo, dental issues, hearing loss, eye/vision problems, and headaches.

**Table 4:**
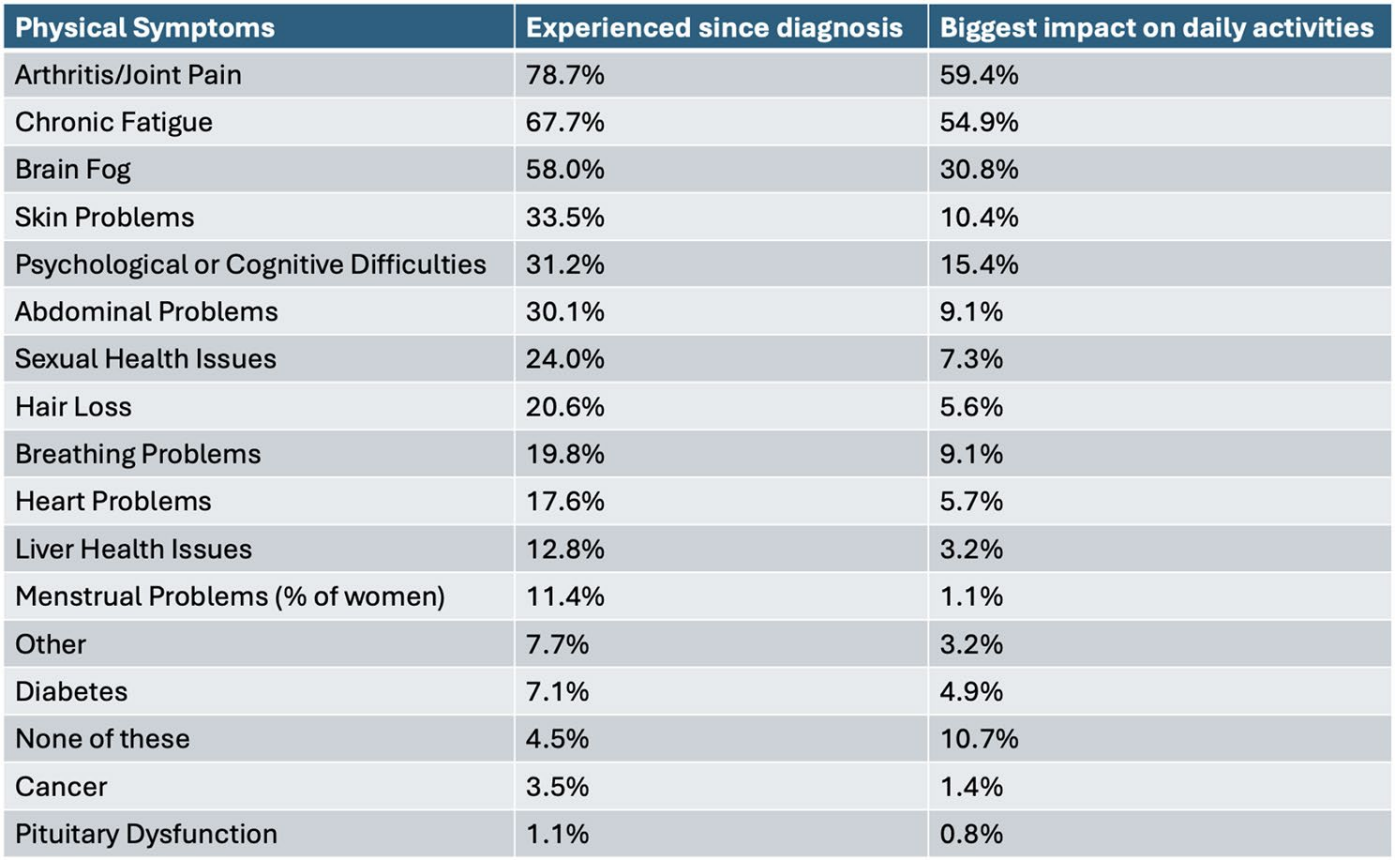
% GH-focussed survey respondents who report experience of each physical symptoms since diagnosis of genetic haemochromatosis and % respondents who report each physical symptom to have the greatest impact on daily activities

**Fig. 1:**
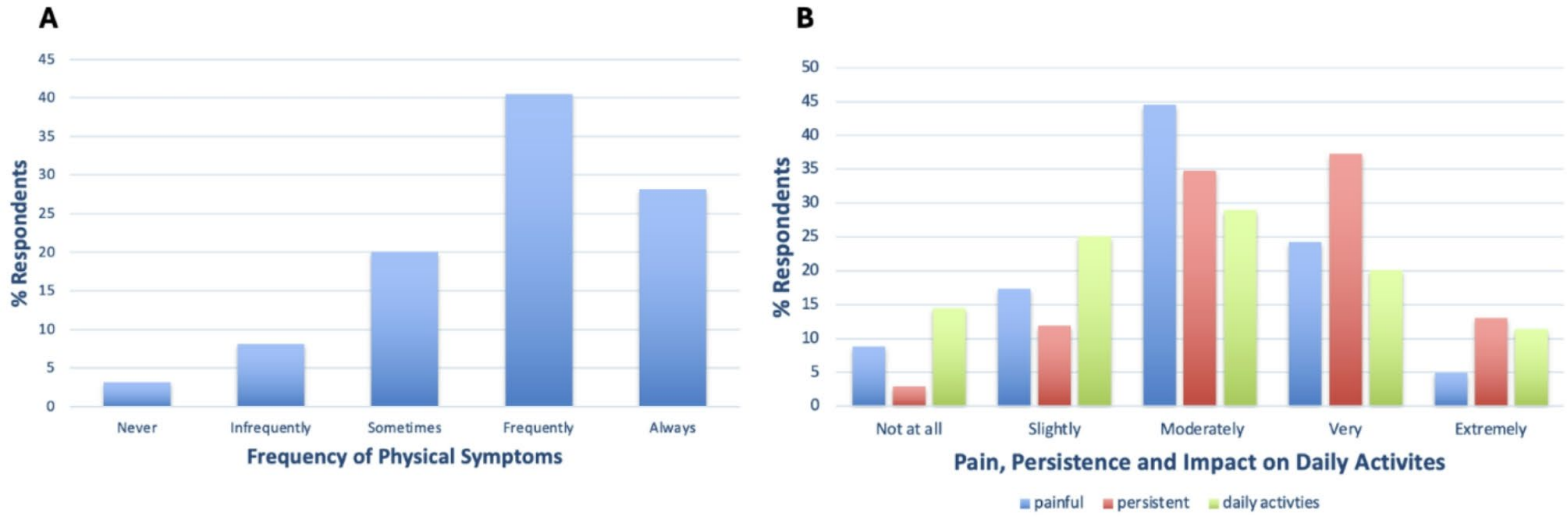
**A** % GH-focussed survey respondents who report having experienced physical symptoms never, infrequently, sometimes, frequently, or always. **B** % GH-focussed survey respondents who report physical symptoms as not at all, slightly, moderately, very, or extremely painful (blue), and persistent (red), and % respondents who report their condition to not at all, slightly, moderately, very, or extremely impact on daily activities (green)

82.6% of those who had experienced a physical symptom reported that they were currently experiencing these symptoms, and symptoms were mainly reported to frequently (40.4%) or always (28.2%) occur (Fig. 1A). Physical symptoms were usually moderately (44.5%) painful and very (37.3%) persistent, but occasionally symptoms extremely painful (5.0%) and persistent (13.1%) (Fig. 1B). Respondents mainly reported a moderate (29.0%) impact of GH on daily activities (29.0%); only 14.4% of respondents reported no impact on daily activities (Fig. 1B).

When respondents were asked how GH impacts daily life, 44.0% experienced elements of fatigue, tiredness, and exhaustion; this was reported to have a negative impact on social life, daily activities, working, and desirable activities such as exercise and hobbies. Joint pain/issues negatively impacted these activities and mobility in 24.6% of respondents. Impact on diet (4.9%), worries about health and future (3.5%), frequent hospital and venesection appointments (1.8%), and worsened symptoms in the period before venesection when iron levels have risen (1.7%), were also reported. Overall, GH can have varying impact on daily activities with some people experiencing “No impact on my life” and others reporting experiences like “fatigue is off the chart and I spend about 20 hours a day in bed” and “have to pace every day activities from decided if can shower or empty dishwasher or make a cup of tea”.

#### Psychological health

Shown in Tables 5, 85.7% of respondents reported experience of at least one mental health symptom in the past month; sleeping problems, low mood, and increased worrying/anxiety were the top symptoms reported. 1.2% selected and other reported symptoms including frustration, social isolation, and anger.

**Table 5:**
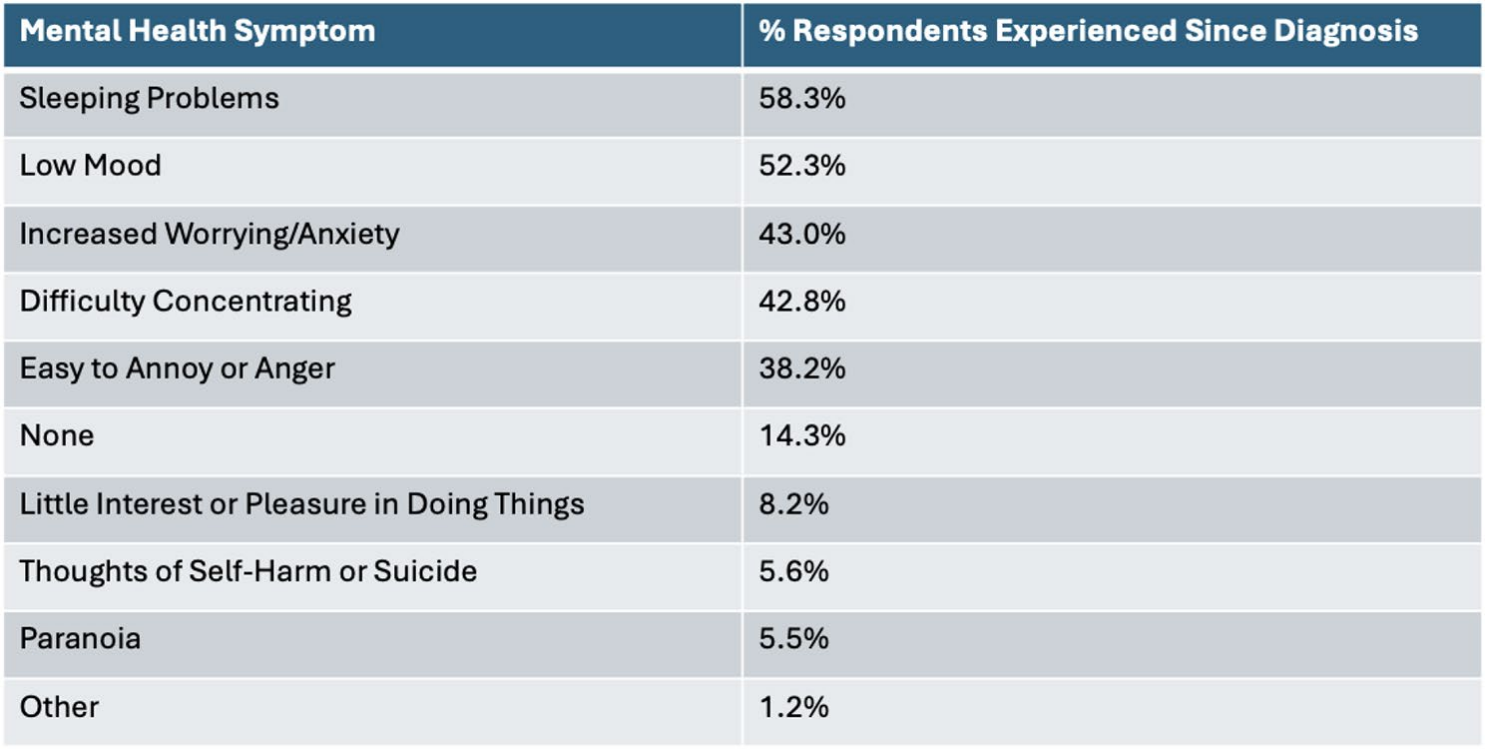
% GH-focussed survey respondents who have experienced each of the listed mental health symptoms in the past month

**Fig. 2:**
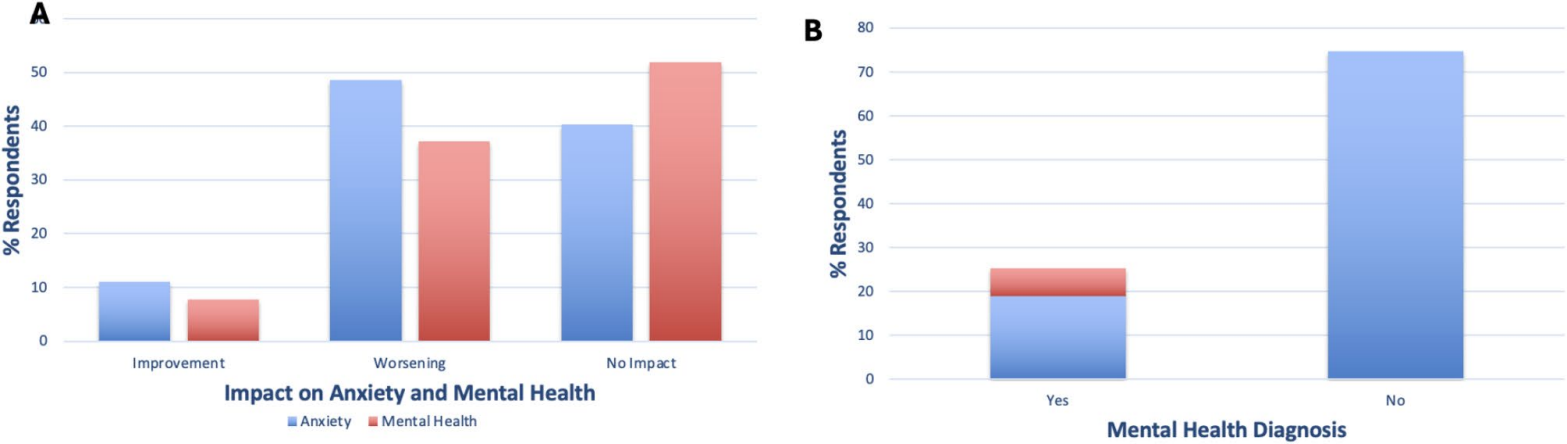
**A** % GH-focussed survey respondents who reported diagnosis of genetic haemochromatosis resulted in an improvement, worsening, or no impact on anxiety (blue) or mental health (red). **B** % respondents who do or do not have a mental health diagnosis, and % respondents who received this diagnosis before (red) or after (blue) diagnosis of genetic haemochromatosis

GH diagnosis had a negative impact on anxiety and mental health for 48.6% and 37.2% of respondents, respectively (Fig. 2A). 25.4% of respondents have received a mental health diagnosis (Fig. 2B); 25.2% of these occured after GH diagnosis. Of the 18.9% of respondents who received mental health support, the majority of these received therapy (47.3%) or drug treatment (36.0%), but smaller percentages reported other (8.6%) or did not provide an answer (8.1%).

### Social relationships, level of independence, work, environment

**Fig. 3:**
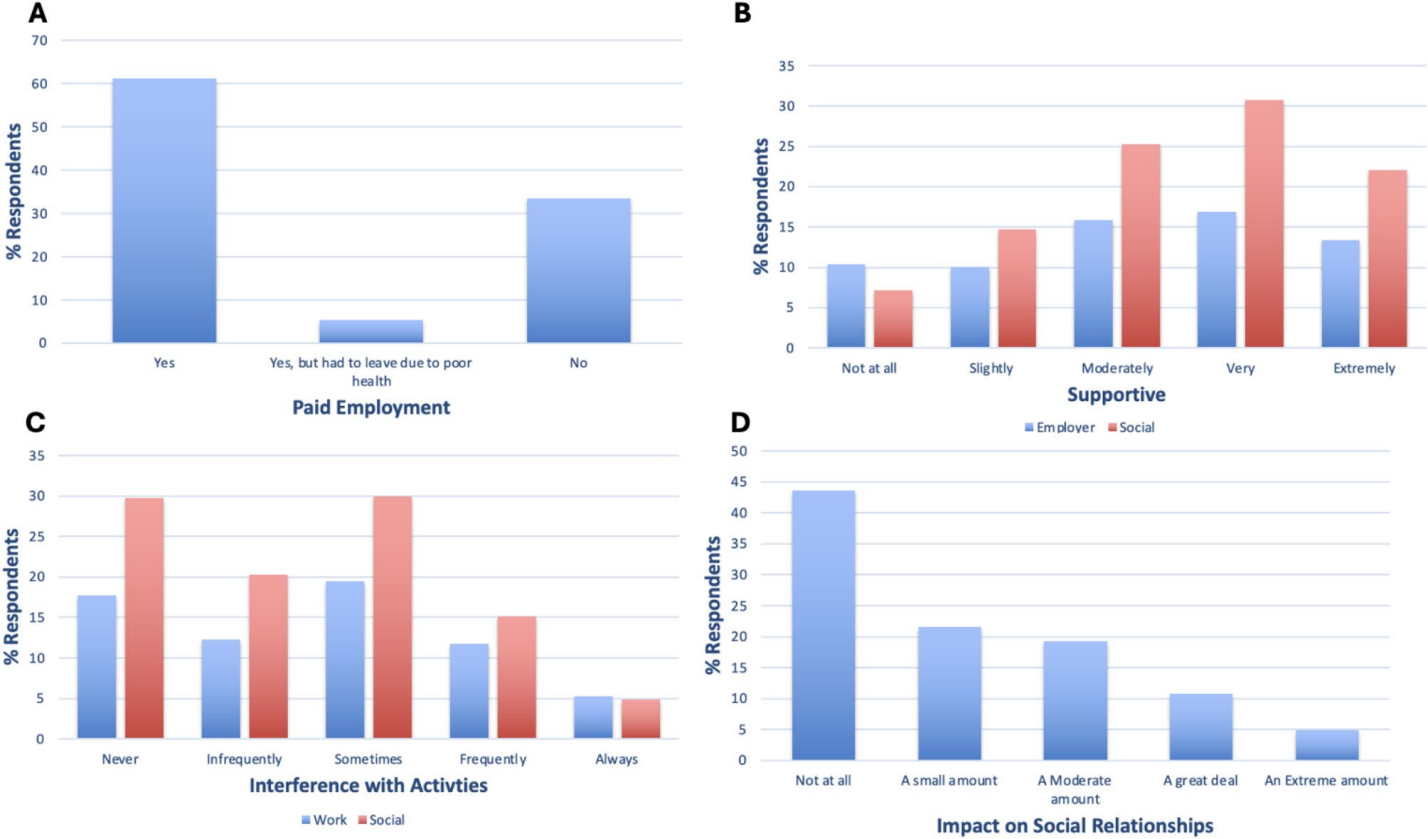
**A** % GH-focussed survey respondents who are and are not in paid employment or had to leave due to poor health. **B** % respondents who had not at all, slightly, moderately, very, extremely supportive employers (blue) or social relationships (red). **C** % respondents who never, infrequently, sometimes, frequently, or always experience interference with work (blue) and social (red) activities. **D** % respondents who report their condition has not at all, a small amount, a moderate amount, a great deal, or an extreme amount of impact on their ability to maintain social relationships

Most respondents were in paid employment (61.2%); however, 5.3% of respondents left work due to poor health (Fig. 3A). 8.5% of respondents reported experience of discrimination due to their condition. Respondents mostly had supportive employers and social relationships but 10.4% and 7.2% respondents reported these were not at all supportive (Fig. 3B). Most respondents reported they experienced some impact of their condition on both work and social activities; only 17.7% respondents reported they never experienced interference with work activities and 29.7% with social activities (Fig. 3C). Top reasons provided for GH interference with work were: fatigue/tiredness (28.0%), brain fog/slow thinking (14.1%), frequent hospital appointments/venesections (9.6%) and joint issues (7.7%). This was also seen in significantly lower opportunities for recreation (F12) score in GH-diagnosed (Table 3). 56.4% of respondents experienced some level of impact on their social relationships due to their condition (Fig. 3D).

#### GH treatment

**Fig. 4:**
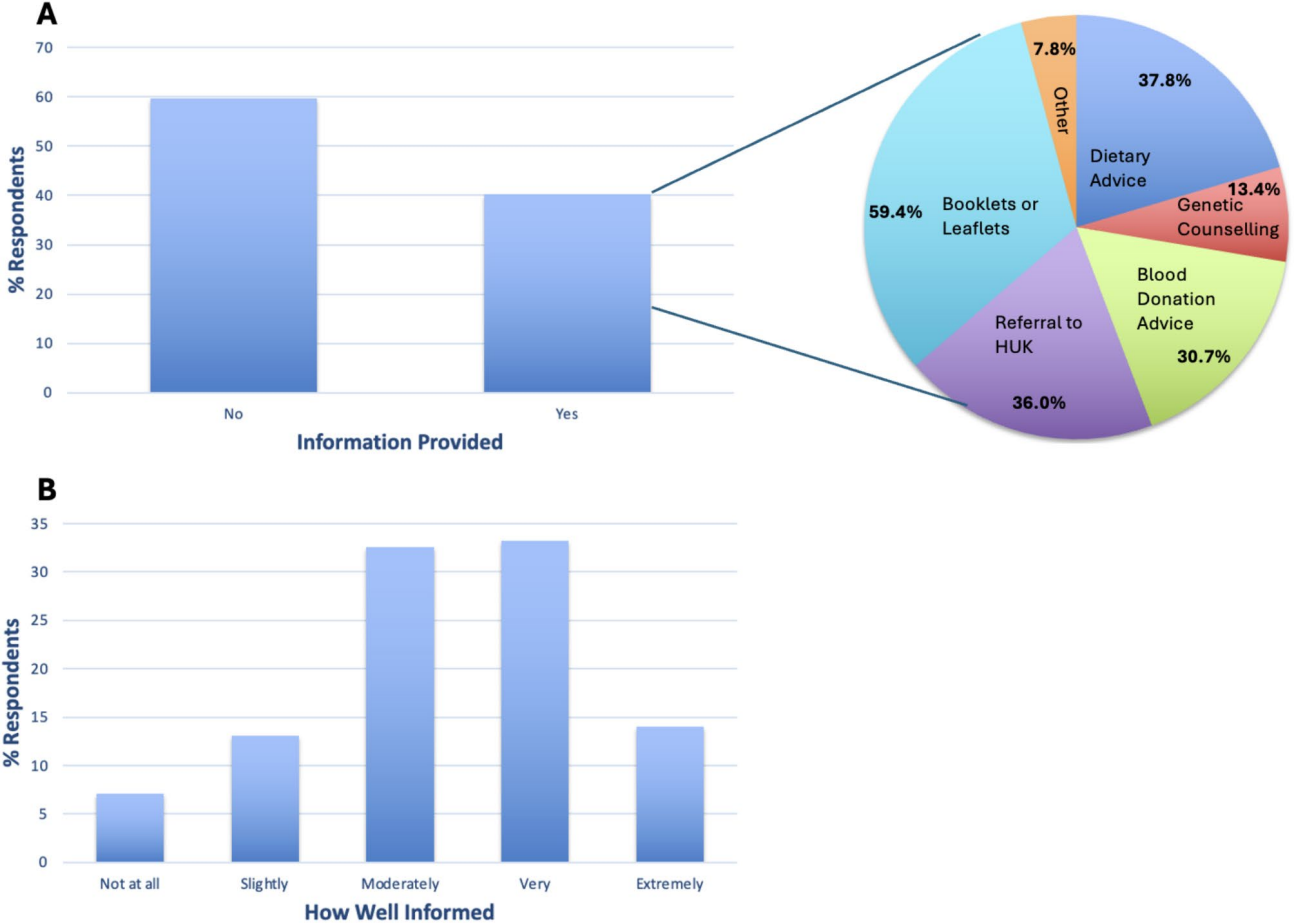
**A** % GH-focussed survey respondents who were provided information on genetic haemochromatosis by an NHS professional, and the types of information provided. **B** % respondents who feel not at all, slightly, moderately, very, or extremely well informed

Only 40.3% of respondents were provided with information from a national health service (NHS) professional following GH diagnosis (Fig. 4A). Those who were provided with information mostly received booklets and leaflets (59.4%), but referrals to websites and general verbal advice was also reported (Fig. 4A). Most (65.8%) respondents felt moderately and very well informed about GH and its management (Fig. 4B).

**Fig. 5:**
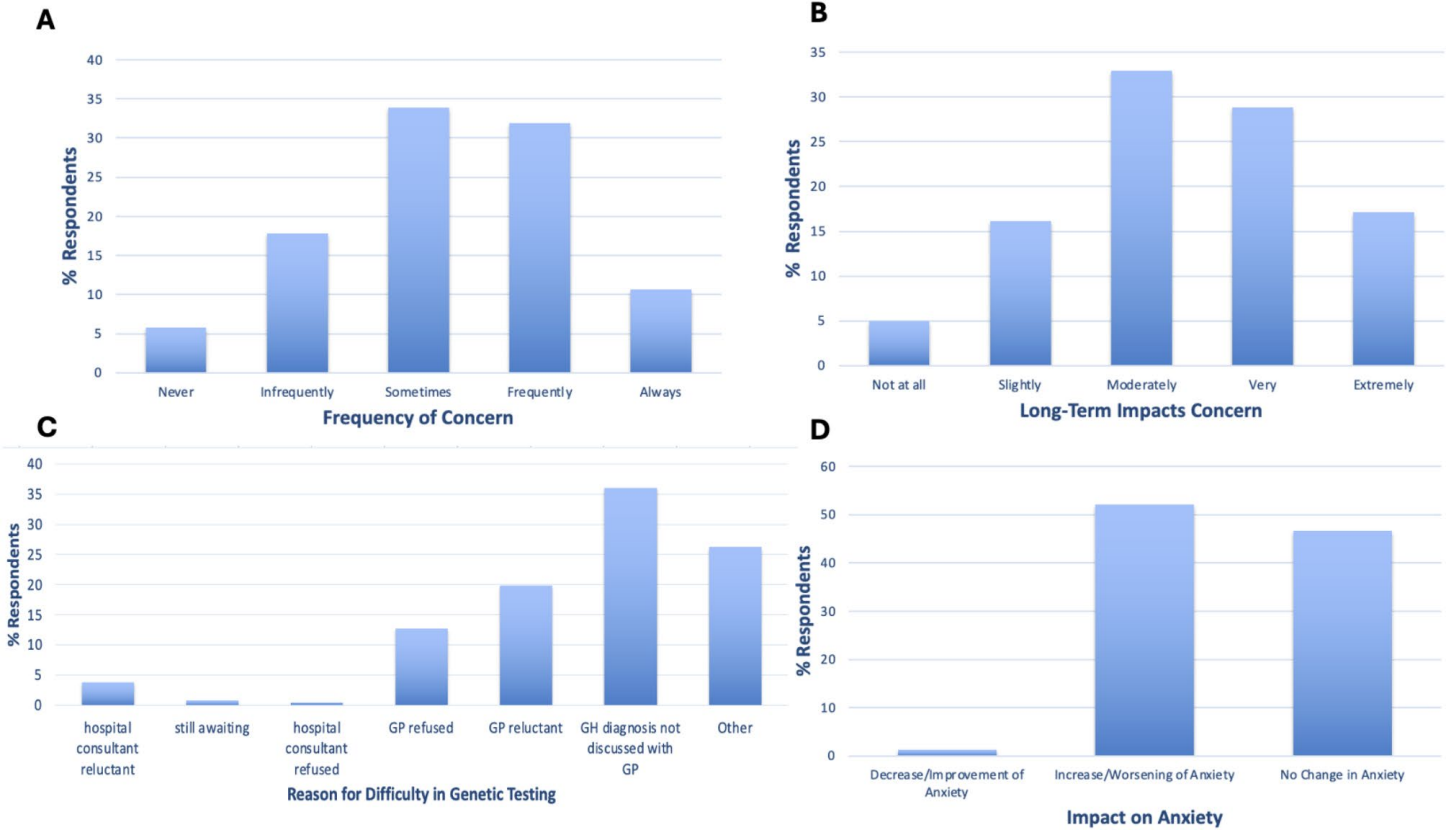
**A** % GH-focussed survey respondents who never, infrequently, sometimes, frequently, or always felt worried or concerned for their health in the past month. **B** % respondent who felt not at all, slightly, moderately, very, or extremely concerned about the long-term impacts of their condition. **C** % GH-focussed survey respondents who report the reason for difficulty obtaining a genetic test as a hospital consultant or GP was refused or was reluctant to order a test; still awaiting test or results, a genetic haemochromatosis diagnosis was not discussed, or other. **D** % respondents who report a decrease, increase, or no change in anxiety as a result of difficulty obtaining a genetic test

Most respondents reported feeling worried or concerned sometimes (33.9%) or frequently (31.9%) for their health in the past month (Fig. 5A). Most respondents also felt moderately (32.9%) or very (28.8%) concerned about long-term impacts of their condition (Fig. 5B).

24.0% of respondents reported difficulty in obtaining a genetic test, with the reasons for this presented in Fig. 5C. Most common reason for difficulty in obtaining a genetic test were lack of discussion of diagnosis with a general practitioner (GP) (36.0%), and GP refusal (12.7%) or reluctance (19.9%) (Fig. 5C). This difficulty resulted in an increase in anxiety in 52.1% of respondents (Fig. 5D).

**Fig. 6:**
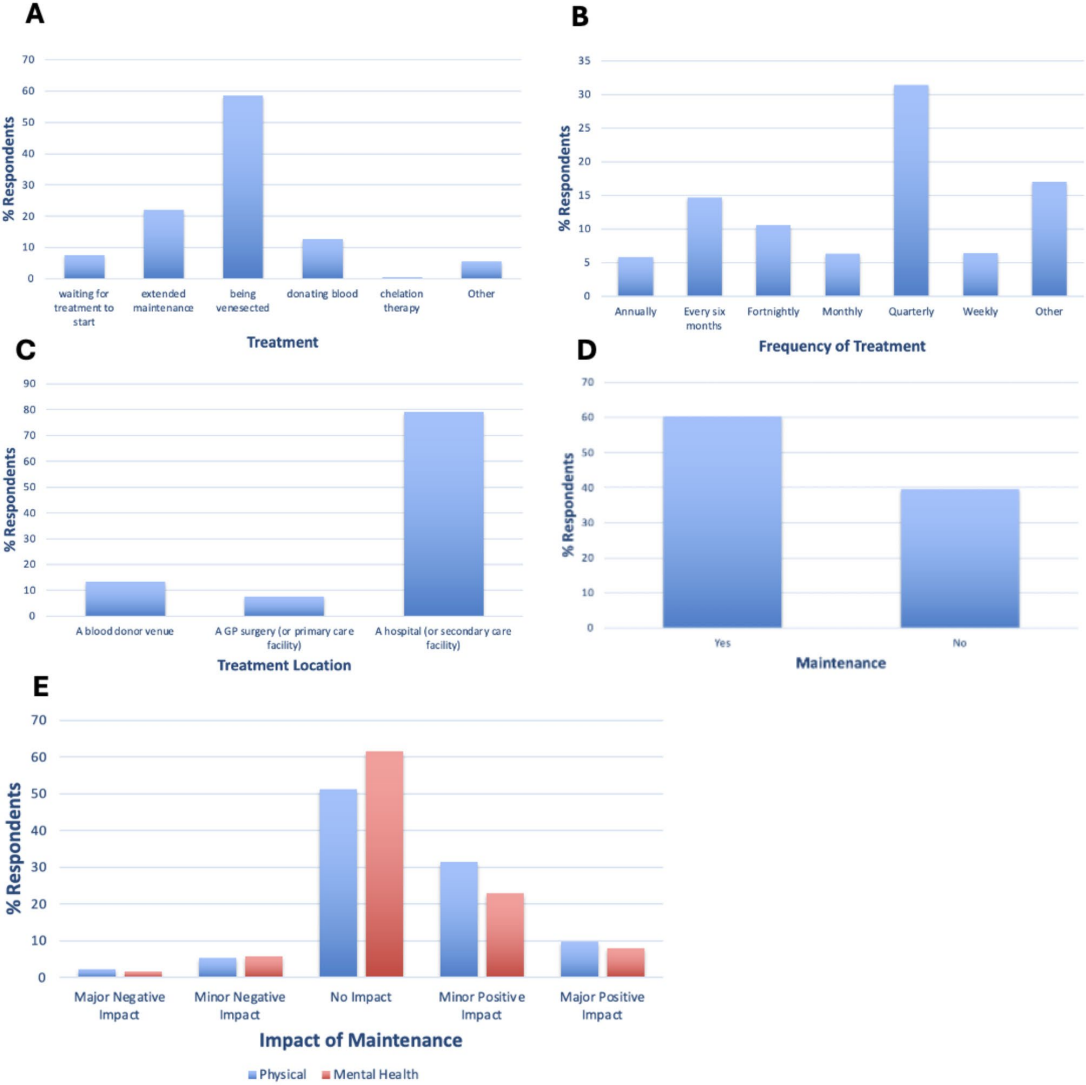
**A** % GH-focussed survey respondents who are waiting for treatment to start, not in active treatment/in extended maintenance, are being venesected, are donating blood (in a blood donor venue), undergoing chelation therapy, or are undergoing other treatment. **B** % respondents who require treatment annually, every six months, fortnightly, monthly, quarterly, weekly, or other. **C** % respondents who receive treatment at a blood donor venue, a GP surgery (or primary care facility), or a hospital (or secondary care facility). **D** % GH-focussed respondents who have reached maintenance. **E** % respondents who have seen major negative impact, minor negative impact, no impact, minor positive impact, or major positive impact on their physical symptoms (blue) and mental health symptoms (red)

The most common form of treatment was venesection (58.5%), but respondents were also waiting for treatment (7.6%), not undergoing active treatment/in extended maintenance (22.1%), donating blood (12.7%), or chelation therapy (0.4%) (Fig. 6A). Treatment was usually given quarterly (31.5%) but treatment frequency varied (Fig. 6B). Treatment was generally received in a hospital (or secondary care facility) (79.1%) (Fig. 6C). Despite high percentages of respondents undergoing treatment, the dependence on medication facet (F11) was significantly lower in GH-diagnosed compared to the healthy sample (Table 3).

39.6% of respondents were in maintenance (Fig. 6D); those who were in maintenance mainly reported maintenance had no impact on physical (51.3%) and mental health (61.6%) symptoms (Fig. 6E). However, a greater number of respondents reported a positive effect for physical (41.3%) and mental health (30.9%) symptoms compared to negative impact on physical (7.5%) and mental health (7.5%) symptoms (Fig. 6E).

**Fig. 7:**
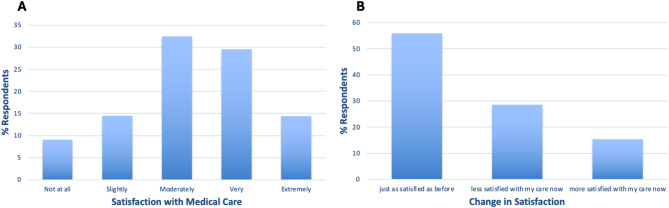
**A** % GH-focussed survey respondents who were not at all, slightly, moderately, very, or extremely satisfied with their medical care. **B** % respondents who were just as satisfied, less satisfied, or more satisfied with their care now compared to before

Most respondents were moderately (32.5%) or very (29.5%) satisfied with their medical care. But 9.0% of respondents were not at all satisfied (Fig. 7A). Respondents were mainly just as satisfied as before (55.9%) with their current care but 28.6% were less satisfied and 15.4% more satisfied (Fig. 7B). Health/Social care (F19) (Table 3) facet was also significantly lower in GH-diagnosed compared to the healthy sample.

Respondents were less satisfied with their medical care due to reduced contact and less attentive healthcare professionals, and difficulty in finding healthcare appointments. Those who felt more satisfied with their medical care reported that their healthcare professionals had improved knowledge of their condition. To improve medical care satisfaction quicker diagnosis, better condition understanding from healthcare professional, less waiting times, more frequent liver testing, more communication, and better more frequent monitoring were reported.

Suggestions of information or support that could improve QoL were: increased and quicker medical care and appointments, in-person appointments rather than virtual or telephone calls, regular checks for organ damage, more dietary advice, and local support. Respondents also reported it would be beneficial if health professionals and others had improved understanding and awareness of GH: one respondent reported “*my GP doesn’t know anything about it and how they can help”.*

## Discussion

### Main findings

The experimental strategy used for this study involved statistical comparison between GH-diagnosed and healthy samples who completed WHOQOL-100. This was followed by a survey specific for GH-diagnosed, to increase understanding for the reasons of QoL rating. The aim of this study was to determine the QoL of GH-diagnosed, and to identify possible reasons for this. The hypothesis was that the QoL in GH-diagnosed would be significantly worse than the QoL of a healthy sample.

The study identified, using WHOQOL-100, that the overall QoL was significantly lower in GH-diagnosed compared to the healthy sample. Evaluation of the domains showed significant reductions in physical, psychological, level of independence, and spirituality domains. Previous studies have not utilised WHOQOL-100 to measure QoL in GH-diagnosed or comparison studies with a healthy sample; there is also a lack of recent studies on QoL in the UK so exact comparisons are not possible. Despite a lack of studies using WHOQOL-100 to measure QoL in GH-diagnosed, use of the Liver Disease Symptom Index, SF-36, and Multi-Dimensional Fatigue Index-20 surveys, found a significantly worse QoL when compared to a Dutch healthy sample [39] and use of Assessment of Quality-of-Life 4D found a decrease in scores compared to a healthy sample [29]. Overall QoL was also shown to be higher in respondents in maintenance, showing it is important for maintenance to be reached. However, this score was still significantly lower than the healthy sample suggesting other methodology may be required to increase QoL.

The significantly lower physical domain score in the GH-diagnosed could be explained by data from the GH-focussed survey, showing experience of various symptoms such as joint pain, chronic fatigue, psychological symptoms etc. These findings agree with previously published data reporting a range of symptoms experienced by GH-diagnosed [11–15]. Additionally, a study which used questionnaires assessing patients habits, fatigue, and joint pain alongside SF-36, found joint pain was one of the biggest factors impacting QoL [3]. The significantly lower psychological domain score in GH-diagnosed, could also be explained by data from the GH-focussed survey suggesting that their condition contributes to worsened mental health; worrying about their condition and long-term impacts, and difficulty in obtaining genetic tests provide possible explanations. The significantly higher environment domain score in those with GH compared to the healthy control could be explained by different distribution of country of residence or income.

Interestingly, healthy scores in overall QoL and domains of this study were lower than that of previous studies [40–44], but this could be explained by the studies being administered in different countries compared to this study in the UK.

### Implications

The GH-focussed survey suggested improvements to healthcare are much needed and may aid in increasing QoL in GH-diagnosed; respondents suggested increased information, access to GPs and other medical care appointments, and better awareness would aid in QoL improvements. The decreased QoL in GH-diagnosed could be addressed through implementation of increased awareness and information of GH for both medical professionals, patients, and the public, alongside better access to medical care and appointments, including quicker diagnosis and treatment to reach maintenance. There are a number of specific findings from our work which are of note to policymakers and healthcare practitioners seeking to improve QoL in GH-diagnosed.

Although reaching maintenance only improved symptoms in 40% of respondents, as most GH-diagnosed were not in maintenance, increasing the number of GH-diagnosed in maintenance may show symptom and QoL improvement in an increased number of patients. The significantly higher QoL score in GH-diagnosed in maintenance compared to those not in maintenance, suggests interventions which expedite progression through treatment to reach maintenance could offer significant benefit to QoL. Treatment could be expedited by increasing frequency of venesection by reducing intervals between phlebotomy sessions. Alternatively, prompt administration of proton pump inhibitors following diagnosis could reduce the need for phlebotomies with HFE-related hemochromatosis [45, 46].

Improvements in satisfaction with medical care could be increased by providing: increased and quicker medical care and appointments; in-person appointments rather than virtual or telephone calls; regular checks for organ damage, more dietary advice; and local support. These changes could address the significantly lower Health/Social care (F19) facet in GH-diagnosed and may aid in improvement of QoL. Improved and quicker healthcare may aid in earlier diagnosis, which could help to reduce the liver damage and cancer which was reported by a high percentage of respondents. As joint pain and fatigue were the physical symptoms which were the most common and had the greatest impact on daily activities, improvements in overall QoL could be secured by focussing on interventions which address these two symptoms.

A high percentages of respondents felt moderately and well informed about GH and its management, this could be because most respondents were HUK members and have received information packages. However, a limited number of respondents were provided with information from a national health service following GH diagnosis, so increased access to patient-centric information support resources could help patients newly diagnosed with GH come to terms with their diagnosis. Respondents reported concerns about the levels of knowledge and understanding of the condition by their primary care providers. Therefore, improving clinician education into the condition and associated care pathways, may contribute to improved QoL for GH-diagnosed patients.

Whilst these specific steps may add practical value to GH-diagnosed patients, care should be taken to ensure that such approaches do not adversely affect QoL. Further research on specific interventions and their propensity to improve QoL in this patient population would be helpful in devising and prioritising QoL-enhancing interventions.

### Limitations

Limitations of this study are that both surveys were self-assessment and did not include clinical evaluation, so symptoms and QoL described is subjective. As the study was advertised online and completed through random sampling, introduction of response bias and will exclusion of individuals without internet access or struggle with internet use may have occured. Additionally, both surveys only considered the participants current QoL, so the full extent of GH on the patients overall QoL overtime was not captured. Interviews may be beneficial to better analyse the impact of GH on QoL which may be difficult to fully explain or understand through a questionnaire. Although, the use of UK WHOQOL-100 has been justified as a reliable in-depth measure of QoL in groups and individual participants [37], long survey length could have led to respondent fatigue, impacting accuracy and reliability of the responses. Another limitation, was the significant difference between the age groups in the two WHOQOL-100 samples; however, the percentage differences were very small, so may not have had an impact. Further study, should ensure matched age and socioeconomic background between GH-diagnosed and healthy groups to ensure any differences are not due to this. The study had a lack of respondents under the age of 30, but this could be explained by diagnosis for HFE (type 1) GH usually occurring over the age of 40 [1, 2, 21].

### Future studies

Future studies to expand on understanding of QoL in GH-diagnosed would be useful to expand knowledge on the impact of disease progression, treatment, and genotype. Other possible further studies are described below. Longitudinal studies to evaluate QoL overtime, with assessment throughout diagnosis, treatment, and maintenance phases, would aid in understanding of how QoL changes with disease progression and management. Evaluation of currently implemented GH treatments/interventions, including venesection, erythrocytapheresis, and chelators, on QoL, could measure effectiveness of current treatments on QoL. Analysis of QoL in each GH genotype would allow identification of any differences in QoL and symptom expression in the different genotypes.

## Conclusions

In conclusion, the QoL in GH-diagnosed is significantly lower than a healthy sample and further research and changes in healthcare are necessary to help increase the QoL. Therefore, the next steps taken should be an increase in awareness and information on GH for both medical professionals, patients, and the public and better access to medical care and appointments including quicker diagnosis and reaching maintenance. These steps may aid to improve QoL in GH-diagnosed.

## Data Availability

The datasets used and/or analysed during the current study are available from Haemochromatosis UK on reasonable request by email to research@huk.org.uk.

## References

[1] Lucas MR et al (2024) HFE genotypes, haemochromatosis diagnosis and clinical outcomes at age 80 years: a prospective cohort study in the UK biobank. BMJ Open 14(3):1–10 10.1136/bmjopen-2023-081926 PMC10936495 38479735

[2] Adams PC (2015) Epidemiology and diagnostic testing for hemochromatosis and iron overload. Int J Lab Hematol 37(Suppl 1):25–30 25976957 10.1111/ijlh.12347

[3] Belhomme N et al (2022) Patient-reported outcomes and their relation with iron parameters in HFE haemochromatosis during maintenance therapy: A prospective cohort study. Liver Int 42(11):2473–2481 35727816 10.1111/liv.15341

[4] Bardou-Jacquet E et al (2017) Worse outcomes of patients with HFE hemochromatosis with persistent increases in transferrin saturation during maintenance therapy. Clin Gastroenterol Hepatol 15(10):1620–1627 28111337 10.1016/j.cgh.2016.12.039

[5] Fonseca PFS et al (2018) Quality of life scores differs between genotypic groups of patients with suspected hereditary hemochromatosis. BMC Med Genet 19(1):1–5 29301508 10.1186/s12881-017-0513-5 PMC5755339

[6] Crawford DHG (2014) Hereditary hemochromatosis types 1, 2, and 3. Clin Liver Dis (Hoboken) 3(5):96–97 30992896 10.1002/cld.339 PMC6448708

[7] Antonello P (2017) Ferroportin disease: pathogenesis, diagnosis and treatment. Haematologica 102(12):1972–1984 29101207 10.3324/haematol.2017.170720 PMC5709096

[8] Acevedo LAU et al (2022) Quality of life scores remained different among the genotypic groups of patients with suspected hemochromatosis, even after treatment period. Genes (Basel) 13(1):1–8 10.3390/genes13010118 PMC8774363 35052458

[9] Pilling LC et al (2019) Common conditions associated with hereditary haemochromatosis genetic variants: cohort study in UK biobank. BMJ 364:1–12 10.1136/bmj.k5222 PMC6334179 30651232

[10] Allen KJ et al (2008) Iron-overload-related disease in HFE hereditary hemochromatosis. N Engl J Med 358(3):221–230 18199861 10.1056/NEJMoa073286

[11] Dr. Kimberley J, Smith PCF-S (2017) Dr. Bridget Dibb and Dr. William Griffiths, Living with the impact of iron overload: Report from a large survey of people with haemochromatosis. pp. 1–28

[12] Barg A et al (2011) Total ankle arthroplasty in patients with hereditary hemochromatosis. Clin Orthop Relat Res 469(5):1427–1435 20665138 10.1007/s11999-010-1483-5 PMC3069280

[13] Duval H et al (2009) Hip involvement in hereditary hemochromatosis: A clinical-pathologic study. Joint Bone Spine 76(4):412–415 19535278 10.1016/j.jbspin.2008.11.012

[14] Fang Z et al (2024) Iron overload promotes hemochromatosis-associated osteoarthritis via the mTORC1-p70S6K/4E-BP1 pathway. Int Immunopharmacol 131:1–15 10.1016/j.intimp.2024.111848 38479156

[15] Jordan JM (2004) Arthritis in hemochromatosis or iron storage disease. Curr Opin Rheumatol 16(1):62–66 14673391 10.1097/00002281-200401000-00012

[16] Akbarialiabad H et al (2024) Dermatologic manifestations of hereditary hemochromatosis: a systematic review. J Eur Acad Dermatol Venereol 39(5):1–11 10.1111/jdv.20098 PMC12023702 38752605

[17] Arora N et al (2024) Ichthyosis skin changes in a patient with hereditary hemochromatosis. Cureus 16(1):1–4 10.7759/cureus.52823 PMC10883895 38406096

[18] Mohammad A et al (2013) High prevalence of fibromyalgia in patients with HFE-related hereditary hemochromatosis. J Clin Gastroenterol 47(6):559–564 23188073 10.1097/MCG.0b013e31826f7ad7

[19] Romero-Cortadellas L et al (2023) Haemochromatosis patients’ research priorities: Towards an improved quality of life. Health Expect 26(6):2293–2301 37503783 10.1111/hex.13830 PMC10632644

[20] Mottelson M et al (2024) Iron, haemochromatosis genotypes, and risk of infections: a cohort study of 142,188 general population individuals. Blood 144(7):693–707 10.1182/blood.2023022235 38728387

[21] Nowak A, Giger RS, Krayenbuehl PA (2018) Higher age at diagnosis of hemochromatosis is the strongest predictor of the occurrence of hepatocellular carcinoma in the Swiss hemochromatosis cohort: A prospective longitudinal observational study. Med (Baltim) 97(42):1–6 10.1097/MD.0000000000012886 PMC6211894 30335010

[22] Tenuta M et al (2024) Iron overload disorders: Growth and gonadal dysfunction in childhood and adolescence. Pediatr Blood Cancer 71(7):1–11 10.1002/pbc.30995 38616355

[23] van Bokhoven MA, van Deursen CT, Swinkels DW (2011) Diagnosis and management of hereditary haemochromatosis. BMJ 342:c7251 10.1136/bmj.c7251 21248018

[24] Abou Yassine A et al (2023) The evolution of Iron-related comorbidities and hospitalization in patients with hemochromatosis: An analysis of the nationwide inpatient sample. Blood Sci 5(2):131–135 37228771 10.1097/BS9.0000000000000151 PMC10205386

[25] Elmberg M et al (2003) Cancer risk in patients with hereditary hemochromatosis and in their first-degree relatives. Gastroenterology 125(6):1733–1741 14724826 10.1053/j.gastro.2003.09.035

[26] Infanti L et al (2024) Blood donation for iron removal in individuals with HFE mutations: study of efficacy and safety and short review on hemochromatosis and blood donation. Front Med (Lausanne) 11:1–14 10.3389/fmed.2024.1362941 PMC10986032 38566922

[27] Rombout-Sestrienkova E et al (2016) Erythrocytapheresis versus phlebotomy in the maintenance treatment of HFE hemochromatosis patients: Results from a randomized crossover trial. Transfusion 56(1):261–270 26358375 10.1111/trf.13328

[28] Kowdley KV et al (2023) Rusfertide for the treatment of iron overload in HFE-related haemochromatosis: An open-label, multicentre, proof-of-concept phase 2 trial. Lancet Gastroenterol Hepatol 8(12):1118–1128 37863080 10.1016/S2468-1253(23)00250-9

[29] de Graaff B et al (2016) Quality of life utility values for hereditary haemochromatosis in Australia. Health Qual Life Outcomes 14:1–9 26922941 10.1186/s12955-016-0431-9 PMC4770680

[30] Prabhu A et al (2020) Systematic review of the clinical outcomes of iron reduction in hereditary hemochromatosis. Hepatology 72(4):1469–1482 32500577 10.1002/hep.31405

[31] Organisation WH (1998) Programme on mental health: WHOQOL User Manual, D.O.M.H.A.P.O.S. ABUSE, Editor

[32] Li L et al (2004) Psychometric properties of the WHO quality of life questionnaire (WHOQOL-100) in patients with chronic diseases and their caregivers in China. Bull World Health Organ 82(7):493–502 15508194 PMC2622901

[33] Bonomi AE et al (2000) Validation of the United States’ version of the world health organization quality of life (WHOQOL) instrument. J Clin Epidemiol 53(1):1–12 10693897 10.1016/s0895-4356(99)00123-7

[34] Masthoff ED et al (2005) Validation of the WHO quality of life assessment instrument (WHOQOL-100) in a population of Dutch adult psychiatric outpatients. Eur Psychiatry 20(7):465–473 16216471 10.1016/j.eurpsy.2004.09.012

[35] Örsel S, Akdemir A, Dağ İ (2004) The sensitivity of quality-of-life scale WHOQOL-100 to psychopathological measures in schizophrenia. Compr Psychiatr 45(1):57–61 10.1016/j.comppsych.2003.09.006 14671738

[36] Den Oudsten BL et al (2009) The WHOQOL-100 has good psychometric properties in breast cancer patients. J Clin Epidemiol 62(2):195–205 18640821 10.1016/j.jclinepi.2008.03.006

[37] Skevington SM (1999) Measuring quality of life in Britain: Introducing the WHOQOL-100. J Psychosom Res 47(5):449–459 10624843 10.1016/s0022-3999(99)00051-3

[38] Skevington SM, Wright A (2001) Changes in the quality of life of patients receiving antidepressant medication in primary care: Validation of the WHOQOL–100. Br J Psychiatry 178(3):261–267 11230038 10.1192/bjp.178.3.261

[39] van der Plas SM et al (2007) Generic and disease-specific health related quality of life of liver patients with various aetiologies: A survey. Qual Life Res 16(3):375–388 17334830 10.1007/s11136-006-9131-y

[40] Ertam I et al (2009) Quality of life and its relation with disease severity in Behçet’s disease. Clin Exp Rheumatol 27(2 Suppl 53):S18–22 19796527

[41] Giray S et al (2009) Health-related quality of life of patients with epilepsy in Turkey. J Clin Neurosci 16(12):1582–1587 19837591 10.1016/j.jocn.2009.03.028

[42] Ozenli Y et al (2008) Health-related quality of life in patients with conversion disorder with seizures. Int J Psychiatry Clin Pract 12(2):105–111 24916620 10.1080/13651500701679379

[43] Tóthová V et al (2014) Quality of life in patients with chronic diseases. Neuro Endocrinol Lett 35(Suppl 1):11–18 25433349

[44] Bicudo NP et al (2016) Quality of life in adults with neurofibromatosis 1 in Brazil. J Genet Couns 25(5):1063–1074 26944915 10.1007/s10897-016-9939-8

[45] Dirweesh A et al (2021) Proton pump inhibitors reduce phlebotomy burden in patients with HFE-related hemochromatosis: A systematic review and meta-analysis. Eur J Gastroenterol Hepatol 33(10):1327–1331 32769410 10.1097/MEG.0000000000001857

[46] Vanclooster A et al (2017) Proton pump inhibitors decrease phlebotomy need in HFE hemochromatosis: Double-Blind randomized Placebo-Controlled trial. Gastroenterology 153(3):678–680e2 28624580 10.1053/j.gastro.2017.06.006

